# A Pilot Study on Covid and Autism: Prevalence, Clinical Presentation and Vaccine Side Effects

**DOI:** 10.3390/brainsci11070860

**Published:** 2021-06-28

**Authors:** Natascia Brondino, Federico Bertoglio, Federico Forneris, Silvia Faravelli, Alessandro Borghesi, Stefano Damiani, Umberto Provenzani, Marta Nola, Miriam Olivola, Monica Caviglia, Pierluigi Politi, Laura Fusar-Poli, Paolo Fusar-Poli

**Affiliations:** 1 Department of Brain and Behavioral Sciences, University of Pavia, 27100 Pavia, Italy; stefano.damiani01@ateneopv.it (S.D.); umbertoprovenzani@gmail.com (U.P.); marta.nola@unipv.it (M.N.); miriamolivola@icloud.com (M.O.); mo.caviglia@gmail.com (M.C.); pierluigi.politi@unipv.it (P.P.); paolo.fusar-poli@unipv.it (P.F.-P.); 2 Abteilung Biotechnologie, Institut für Biochemie, Biotechnologie und Bioinformatik, Technische Universität Braunschweig, 38106 Braunschweig, Germany; f.bertoglio@tu-braunschweig.de; 3 Armenise-Harvard Laboratory of Structural Biology, Department of Biology and Biotechnology “Lazzaro Spallanzani”, University of Pavia, 27100 Pavia, Italy; federico.forneris@unipv.it (F.F.); silvia.faravelli01@universitadipavia.it (S.F.); 4 Neonatal Intensive Care Unit, Fondazione IRCCS Policlinico San Matteo, 27100 Pavia, Italy; a.borghesi@smatteo.pv.it; 5 Cascina Rossago, San Ponzo Semola, 27100 Pavia, Italy; 6 Department of Clinical and Experimental Medicine, University of Catania, 95124 Catania, Italy; laura.fusarpoli@gmail.com; 7 Psychology & Neuroscience, Department of Psychosis Studies, Institute of Psychiatry, King’s College London, London WC2R 2LS, UK; 8 OASIS Service, South London and Maudsley NHS Foundation Trust, London SE11 5DL, UK

**Keywords:** autism spectrum disorders, COVID-19, susceptibility, antibody response, vaccine

## Abstract

*Background*: Several neurobiological mechanisms have been proposed to support the hypothesis of a higher COVID-19 risk in individuals with autism spectrum disorder (ASD). However, no real-world data are available on this population. *Methods*: We compared the period prevalence (March–May 2020) and symptom presentation of COVID-19 infections between a sample of individuals with severe ASD (*n* = 36) and the staff personnel (*n* = 35) of two specialized centers. Anti-SARS-Cov-2 antibody positivity was used as a proxy of infection. Additionally, we evaluated vaccine side effects in the same groups. *Results*: No significant difference was found between the prevalence of COVID-19 positivity between autistic participants and staff personnel. Levels of antibodies against the spike protein and the receptor binding domain were not significantly different between autistic and staff participants. The level of antibodies against the N-terminal domain were higher in autistic individuals. There was a significant difference between the prevalence of symptomatic COVID-19 in autistic participants (9.1%) compared to staff personnel (92.3%). The most frequent side effect among autistic participants was light fever. *Conclusions:* The present study provides preliminary data on COVID-19 transmission and presentation in ASD. Our data do not support the hypothesis of a higher susceptibility and severity of COVID-19 in people with ASD.

## 1. Introduction

The COVID-19 pandemic has disrupted the lives of billions of people, adding uncertainty, and causing psychological distress to the world population. Individuals with autism spectrum disorder (ASD) represent a population potentially more vulnerable in the time of a pandemic. Autistic individuals of all ages, as well as their caregivers, have been considerably impacted by stay-at-home orders, routine disruptions, and new social distancing standards [1]. Additionally, ASD has been proposed as a potential risk factor for the development of COVID-19 infections. In fact, autistic individuals, especially in the presence of a comorbid intellectual disability (ID), may experience difficulties in maintaining social distancing and in using personal protective equipment (PPE) correctly due to sensory difficulties. Moreover, literature data point out that autistic people are more prone to both bacterial and viral infections [2]. Specific ASD pathophysiological mechanisms have been advocated as potential risk factors for COVID-19 [3]. First, the presence of an altered immune response, characterized by an increase in pro-inflammatory cytokines and impairment in cellular-mediated response, has been frequently reported in ASD [4,5,6]. Second, atypical antipsychotics, such as risperidone, frequently prescribed to treat irritability in ASD [7], have been shown to exert anti-inflammatory effects and disrupt both innate and adaptive immune responses [8] and thus represent an adjunctive risk factor for COVID-19 in the autistic population. Third, six genes previously associated with ASD have been demonstrated to be differentially regulated in individuals with severe COVID-19 [9]. Finally, several ASD individuals showed melatonin deficiency or alteration in the melatonin genes: as the melatonin system acts both as an immune regulator and an oxidant scavenger, impairment in this system has been hypothesized to impact COVID-19 susceptibility in autistic people [10]. Despite these premises, real-world data on COVID-19 prevalence among autistic individuals are lacking. To our knowledge, only two papers have been published on the topic: the first one was a case report describing a high viral load in an autistic child suffering from COVID-19 in a residential facility [11]; the second case series considered the characteristics of infection among autistic residents of a neuropsychiatric facility which was transformed into a COVID-19 ward and did not provide data on COVID-19 infections in a control group, such as staff personnel [12].

The aims of the present study are: (a) to assess the susceptibility of autistic patients to COVID-19 by comparing the prevalence rates of COVID-19 in autistic patients and staff of two Italian facilities specifically dedicated to autistic adolescents and adults; (b) to evaluate the clinical presentation of COVID-19 in autistic patients and staff; (c) to record the side effects of COVID-19 vaccinations in the same population.

## 2. Materials and Methods

### 2.1. Setting

The study of natural infection by SARS-CoV-2 was conducted between March 2020 and May 2020 in two centers: the daycare center “Il Tiglio” and the farm community “Cascina Rossago”, which are a day center and a residential facility specifically designed for individuals with severe ASD and comorbid ID, located in the Lombardy Region, Italy, near two regional nature parks.

The daycare center “Il Tiglio” accommodates 18 individuals, and the daily staff is composed of 7 therapists, 1 psychologist, 1 kinesiologist, and 2 care assistants. Constant contact between ASD individuals and staff was present for two weeks before the general lockdown of the country (9 March 2020), during which several accommodations were made. However, PPE was not provided for both staff and ASD subjects, who were in close proximity with a high risk of infection transmission. The daycare center was closed after the promulgation of lockdown restrictive measures. Immediately after closure, almost all the staff developed symptoms consistent with COVID-19 infection and, after two weeks, the same happened in some families of autistic patients. Therefore, all autistic individuals in this setting could be considered exposed to SARS-CoV-2. The daycare center reopened at the end of May 2020 and, before reopening, each subject and staff member underwent a blood sample in order to determine the presence of SARS-CoV-2 antibodies according to the standard Italian National Health System (NHS) determination.

The farm community “Cascina Rossago” accommodates 22 adults (17 males and 5 females), and the daily staff is composed of 17 therapists, 3 nurses, and 4 care assistants. The farm community was closed to external visits between March 2020 and May 2020, but staff members were allowed to work in the facility during the lockdown period. However, due to the imposed restrictions, they were not allowed to leave their homes if not for work or urgent reasons (e.g., groceries). Therefore, the contact with potential sources of infections was essentially limited to the farm community. Similar to the daycare center, nurses and some staff members developed symptoms consistent with COVID-19 after the beginning of the lockdown (March–April 2020). Autistic individuals due to sensory issues were not able to attune to the use of PPE (while all staff started to wear PPE as soon as it was provided) and they lived all together as in a household. Therefore, all autistic subjects could be considered exposed to SARS-CoV-2. At the end of May 2020, each subject and staff member underwent a blood sample in order to determine the presence of anti-SARS-CoV-2 antibodies according to the standard Italian NHS determination.

### 2.2. Procedures

All autistic subjects were diagnosed according to the fifth edition of the Diagnostic and Statistical Manual of Mental Disorders (DSM 5) criteria by a senior psychiatrist with wide expertise in ASD. Staff were used as a natural control group, as this group could be considered exposed to the same conditions and environment as the autistic participants.

Collections of COVID-19 symptoms and blood sampling were performed in May 2020, as this was required for reopening in the case of the daycare center, or for control purposes in the case of the farm community.

The study of vaccine side effects was conducted in March 2021, when patients and staff were all fully vaccinated with the Comirnaty vaccine.

**Figure 1:**
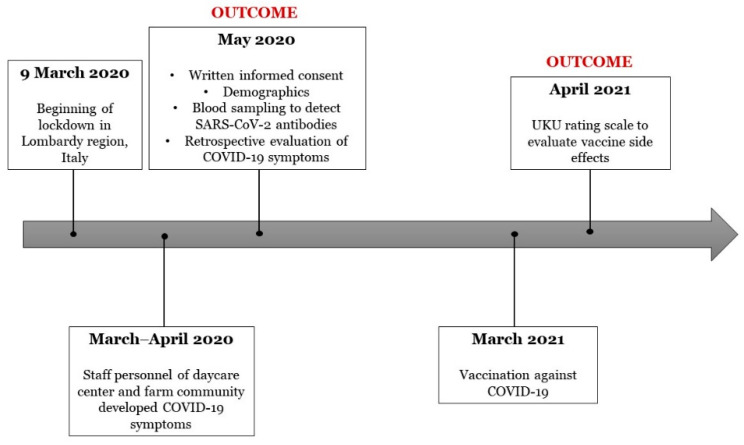
Timeline of the study procedures.

The timeline of study procedures has been depicted in Figure 1.

Written informed consent was provided by patients or their legal representatives before entering the study. The protocol was approved by the Ethics Committee of IRCCS Policlinico San Matteo, Pavia, Italy (P_20200057015 of 09/07/2020).

### 2.3. Blood Sampling and Detection of Antibodies Levels

Blood samples were drawn from the antecubital vein in the morning between 9:00 and 11:00 a.m. Serum was immediately extracted, aliquoted and stored at −80 °C until the analysis. The assays for detection of SARS-CoV-2 antibodies were conducted independently by our NHS laboratory and in two different university laboratories.

To detect specific antibodies in university laboratories, recombinant SARS-CoV-2 spike receptor binding domain (RBD) was produced using polyethyleneimine-based transient transfection of Freestyle HEK293 Cells (Life Technologies) cultivated in suspension according to Faravelli and colleagues [13]. The pCAGGS plasmids for production of the C-terminal His-tagged SARS-CoV-2 Spike RBD (#NR_52310) were obtained from BEI Resources (NY, USA). Serum was tested for the presence of IgA and IgG against the receptor binding domain of the SARS-CoV-2 spike protein by means of the ELISA-based assay as described in Bruni et al. [14]. The method has excellent specificity (86%) and sensitivity (97%) for IgGs and proved suitable for the detection of anti- SARS-CoV-2 IgAs with excellent sensitivity and specificity as well. Briefly, the SARS-CoV-2 spike RBD was diluted in phosphate buffered saline (PBS tablets E404-200TABS, VWR) at a final concentration of 2 µg/mL, and 50 µL of solution was used to coat 96-well plates (Nunc MaxiSorp™ flat-bottom, ThermoFisher, Waltham, MA, USA) overnight at 4 °C. The day after, the coating solution was removed, and the plates were washed thrice with 200 µL of 0.1% of Tween-20 (Sigma, Kawasaki, Japan, P1379) in PBS (hereafter, called PBST). A total of 200 µL of 3% bovine serum albumin (BSA, Sigma, Kawasaki, Japan, A7030) diluted in PBS (blocking solution) was added to each well for at least 1 h at room temperature. In the meantime, samples were centrifuged at 4000 rpm for 10 min at room temperature and then diluted 1:50 and 1:200 with 1% BSA in PBST (hereafter, reagent solution). After 1 h of incubation, the blocking solution was removed and the plates washed as described above, then 50 µL of the diluted samples was plated and incubated for 1½–2 h at room temperature. After the incubation step, the plates were again washed, and 50 µL of a 1:3000 dilution of mouse anti-human IgG-horseradish peroxidase (HRP) conjugated secondary antibody (BD, clone G18-145) or a 1:12,000 dilution of HRP-conjugated donkey anti-human IgA (Biolegend, San Diego, CA, USA, clone Poly24110), both prepared in reagent solution, was added to the plate and incubated for 1 h at room temperature. Next, the plates were washed and 50 µL of tetramethylbenzidine substrate reagent (Sigma, Kawasaki, Japan, T0440, ready to use) was added. The reaction was stopped after 10 min with 50 µL H_2_SO_4_ 1N and the absorbance was measured at 450 nm in a Glomax plate reader. According to Bruni et al. [14], the threshold lines for IgG and IgA were set as 0.277 and 0.295, respectively. 

Additionally, for validation of positive hits emerging from the initial serological investigations, IgG levels against different subdomains belonging to the ectodomain of the spike protein of SARS-CoV-2 were evaluated using ELISA-based immunodetection. Antibody titers against the following spike subdomains (S1-S2 with D614G mutation -res. 14-1208-, N-terminal domain (NTD) –res. 14-303-, RBD-SD1 -res. 319-591-, and S2 -res. 686-1208-) were measured by means of ELISA-based immunosampling starting from an initial serum dilution of 1:314. Measurements were conducted in 384-well plates as described in Bertoglio et al. [15], as well as antigen cloning, production, and purification.

### 2.4. Questionnaires

An ad-hoc questionnaire evaluating COVID-19 symptoms was completed for each participant and a review of medical charts of each autistic participant of the previous three months before blood sampling was conducted.

After patients and staff were all fully vaccinated with the Comirnaty vaccine, the UKU side effect rating scale [16] for adverse events was completed for each participant.

### 2.5. Statistical Analysis

Continuous variables were not normally distributed and thus data were presented as medians and interquartile ranges (IQR). Categorical variables were presented as percentages and counts. Differences in period prevalence rates and symptoms (fever, cough, diarrhea, pneumonia/dyspnea, hospitalization) between patients and staff as well as differences between the daycare center and the farm community’s autistic participants, were evaluated by means of a Fisher’s exact test or Chi-squared test, as appropriate. The difference between autistic individuals taking antipsychotics and subjects not on antipsychotic medications was determined by means of a Chi-squared test. The half maximal effective concentration (EC50) determination of IgG titration curves was determined with the ELISA plate reader (Epoch, BioTek, BioTek Instruments, Winooski, VT, USA) software Gene Five 3.03. any difference in antibodies titers between patients and staff was evaluated by means of a Mann–Whitney test. A two-tailed *p*-value < 0.05 was regarded as statistically significant. IBM SPSS 23.0 (IBM Corp., Armonk, NY, USA) was used for all calculations.

## 3. Results

General characteristics of the study participants for each center are reported in Table 1. Fourteen autistic individuals in the daycare center and 22 in the farm community agreed to participate. Eighteen staff subjects agreed to participate in the determination of IgG and IgA antibodies (*n* = 10 for the daycare center and *n* = 8 for the farm community), while all agreed (*n* = 11 for the daycare center and *n* = 24 for the farm community) to report about COVID-19 positivity (NHS measurement), symptoms, and vaccine side effects.

### 3.1. Susceptibility to SARS-CoV-2 Infection

**Figure 2:**
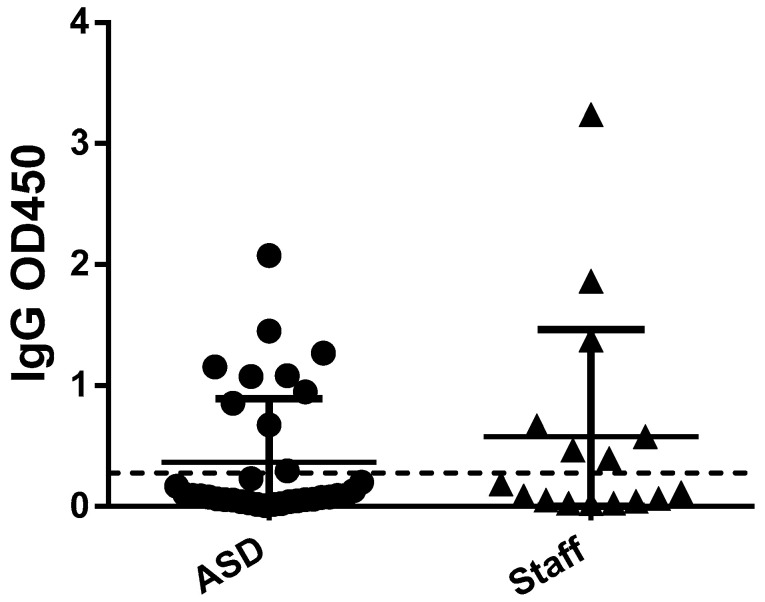
Difference in IgG level between ASD subjects and staff.

Considering the entire sample, there was no significant difference between the prevalence of NHS COVID-19 positivity between autistic participants and staff personnel (Chi-square = 0.71, *p* = 0.39). The same effect was applied also to SARS-CoV-2 IgG positivity (Chi-square = 2.60, *p* = 0.10) (Figure 2) and IgA positivity (Chi-square = 0.60, *p* = 0.44). Levels of antibodies were generally low in the analyzed samples (LogEC50 below 3). In particular, antibodies against the spike protein and the RBD were not significantly different between autistic and staff participants (U = 25, *p* = 0.49; U = 27, *p* = 0.63, respectively). Level of antibodies against the NTD domain were significantly different between autistic participants and staff personnel (U = 16, *p* = 0.05), being higher in autistic individuals. No significant difference in NHS COVID-19 positivity was detected between participants taking antipsychotics or not (Chi-square = 0.01, *p* = 0.9).

At the daycare center, NHS antibody positivity for COVID-19 was present in 81.8% of the staff personnel and in 42.8% of the autistic subjects. The difference between the two groups was statistically significant (Chi-square = 3.75, *p* = 0.05). At the farm community, NHS antibody positivity for COVID-19 was detected in 20% of the staff personnel and in 13.6% of the autistic subjects. The difference between the two groups was not statistically significant (Chi-square = 0.33, *p* = 0.57). At the daycare center, SARS-CoV-2 IgG positivity was present in 81.8% of the staff personnel and in 42.8% of the autistic subjects. At the farm community, SARS-CoV-2 IgG positivity was detected in 12.5% of the staff personnel and in 18.1% of the autistic subjects. At the daycare center, SARS-CoV-2 IgA positivity was present in 18.1% of the staff personnel and in 7.1% of the autistic subjects. At the farm community, SARS-CoV-2 IgA positivity was detected in 12.5% of the staff personnel and in 0.9% of the autistic subjects.

### 3.2. Clinical Presentation

There was a significant difference between the prevalence of symptomatic COVID-19 in autistic participants and staff personnel (9.1% vs. 92.3%, Chi-square = 19.3, *p* < 0.001). Additionally, while two cases of severe COVID-19 pneumonia requiring hospitalization were present in staff personnel (both females, one 41 years old and the other 40 years old), no cases were present among autistic participants. Of note, among the ten autistic participants showing positivity to COVID-19, only one presented a fever (max 38 °C) for two days and cough for one week, not requiring medications.

### 3.3. Vaccine Side Effects

The most frequent side effect among autistic participants was light fever (37.5 °C) (*n* = 7), followed by fatigue (*n* = 2). One autistic participant showed a worsening in problem behaviors for a week following vaccination. The most frequent side effect among staff participants was fatigue and light fever (<37.5 °C) (*n* = 14), which determined 18 lost workdays. All staff participants experienced pain at the site of injection.

## 4. Discussion

Despite the growing number of hypotheses on the potential higher risk for COVID-19 infection among autistic individuals [3,10], we did not observe a higher rate of infection among ASD subjects compared to staff personnel. Although the difference in total prevalence rate was not statistically significant, in the daycare center almost all of the staff acquired the infection at the beginning of the pandemic, as detected through antibody positivity and consistent with symptom development; meanwhile, less than half of the autistic subjects had antibody positivity to SARS-CoV-2. This result may be explained by the inability of autistic subjects to produce specific IgG antibodies or, alternatively, may indicate a natural resistance to the acquisition of a SARS-CoV-2 infection. Additionally, in the farm community the number of infected autistic individuals was very low considering that patients were unable to wear PPE and prone to be in close proximity to each other, as happens in every household. Moreover, the younger age of autistic patients should have conferred a higher susceptibility [17].

ASD subjects displayed a more favorable clinical presentation compared to staff personnel. This could be partly due to a younger age in the autistic population compared to staff [18]. However, the low rate of infection together with a silent clinical presentation could at least raise the hypothesis that ASD may provide a sort of protection against the acquisition of SARS-CoV-2 infection, instead of an additional risk. This is in contrast with the higher rate of infection usually observed in this patient population. It could be hypothesized that early and frequent infections [2], such as influenza and the common cold, could have primed the immune system to have a more adequate response to a novel viral threat. Additionally, it has been hypothesized that the presence of higher levels of interferon-gamma, displayed by ASD subjects [19], could be protective against COVID-19 [20]. Furthermore, the presence of a pro-inflammatory status [21,22] in ASD could be protective against the cytokine storm which is believed to determine severe COVID-19 symptoms [23]. Our results are in line with a recent pre-print meta-analysis [24] which did not observe an increased mortality or risk for severe COVID-19 in patients with developmental disabilities.

COVID-19 vaccine administration was not associated to significant side effects in autistic participants, who, in general, showed a better response compared to staff. This topic is of particular importance, given the fact that several parents of autistic individuals, despite the evidence of a non-association between ASD and vaccines [25], are still extremely reluctant to provide consent for vaccination. In our sample, three subjects did not receive a COVID-19 vaccine due to consent denial from the parents. Additionally, prioritizing vaccination in people with severe mental illness is both a health and political issue [26,27], and in Italy vaccination for people with mental disorders has been scattered and unequal between different regions.

The present study should be interpreted in light of several limitations. First, it is a pilot study which included a small sample. Case-control studies with larger sample sizes may help clarify the actual susceptibility of the autistic population to COVID-19 infection, as well as the clinical presentation of symptomatology, and the vaccination side effects compared to the neurotypical population. Second, we chose a naturalistic control group composed of staff members of the daycare center and the farm community in which autistic participants were accommodated. This was not only due to logistic reasons, but mainly to the shared environmental conditions of the two groups, which could be considered similarly predisposed to COVID-19 infection. Nevertheless, is important to underline that autistic individuals were confined in the farm community or had limited social activities outside of the daycare center; on the contrary, in spite of the stay-at-home order issued by the Italian government during the lockdown period (March–May 2020), the staff personnel may have been in contact with more sources of infection. Third, given the small sample size, we could not take into account potential confounding factors such as age and sex, which may have had an impact on infection susceptibility and vaccine side effects [17]. Finally, as our sample was composed of people with severe ASD and associated ID, we cannot generalize our findings to the higher-functioning part of the spectrum.

## 5. Conclusions

The present study provides preliminary data on COVID-19 prevalence and clinical presentation, as well as vaccine side effects, in people with ASD. Our promising findings advocate for a paradigm shift in the conception of ASD, which should be considered not only as a vulnerable population in need of protection but also as a group with unsuspected strengths needing further investigation.

## Figures and Tables

**Table 1:** General characteristics of the sample.

Baseline Characteristic	ASD Participants (*n* = 36)		Staff Participants (*n* = 35)			ASD Daycare Center (*n* = 14)		ASD Farm Community (*n* = 22)		
	Median or n	IQR or %	Median or n	IQR or %	*p*-Value	Median or n	IQR or %	Median or n	IQR or %	*p*-Value
Sex										
Female	8	22.2	25	71.4	<0.001	2	14.3	6	27.3	0.44
Male	28	77.8	10	28.6		12	85.7	16	72.7	
Age	29.5	24–40.75	38	28–48	0.01	24	18.75–27.25	38.5	29–49	<0.001
COVID-19 NHS positivity	10	27.8	15	37.1	0.39	6	42.8	4	18.1	0.14
COVID-19 Ab positivity ^a^										
IgG	10	27.8	9	50	0.10	6	42.8	4	18.1	0.14
IgA	3	8.3	3	16.7	0.44	1	7.1	2	0.9	1
Use of antipsychotics	21	58.3	-	-	-	10	71.4	11	50	0.20
COVID-19 symptoms										
Fever	1	9.1	12	92.9	<0.001	1	7.1	0	0	0.39
Cough	1	9.1	12	92.9	<0.001	1	7.1	0	0	0.39
Diarrhea	0	0	0	0	-	0	0	0	0	-
Pneumonia/dyspnea	0	0	2	15.4	0.23	0	0	0	0	-
Hospitalization	0	0	2	15.4	0.23	0	0	0	0	-

Note: Percentage of COVID-19 symptoms were calculated on the sample displaying NHS positivity (*n* = 10 for the ASD sample and *n* = 15 for the staff sample). ^a^ COVID-19 positivity was tested in all the autistic samples and in 17 staff subjects.

## Data Availability

The data presented in this study are available on request from the corresponding author.
